# Micro- and nano-encapsulated metal and alloy-based phase-change materials for thermal energy storage

**DOI:** 10.1039/d0na01008a

**Published:** 2021-06-14

**Authors:** Shilei Zhu, Mai Thanh Nguyen, Tetsu Yonezawa

**Affiliations:** Division of Materials Science and Engineering, Faculty of Engineering, Hokkaido University Kita 13 Nishi 8, Kita-ku Sapporo Hokkaido 060-8628 Japan tetsu@eng.hokudai.ac.jp

## Abstract

An overview of recent literature on the micro- and nano-encapsulation of metallic phase-change materials (PCMs) is presented in this review to facilitate an understanding of the basic knowledge, selection criteria, and classification of commonly used PCMs for thermal energy storage (TES). Metals and alloys with high thermal conductivity can be used as PCMs for rapid heat storage in compact systems owing to their high volumetric TES density. The emerging application of metal PCMs in different fields such as solar thermal energy management, smart wearable devices with thermal comfort control, and cooling of electronic devices call for the need of micro- and nano-TES particles, which can be synthesised in different forms to satisfy specific requirements. As metals are easily oxidised, especially at the micro- and nano-level, encapsulation of metal-based PCM particles is important for sustainable use at high operating temperature in ambient conditions. Recent studies focusing on the encapsulation of metallic PCMs at the micro- and nano-level have been reviewed and classified in terms of the melting point of metal/alloy PCMs used and types of encapsulation materials, such as oxides, polymers, carbon, and metals. The current review is expected to provide an outlook on novel metal and alloy PCMs with function-directed structures and superior TES properties for a broad range of applications.

## Abstract 2

A new class of phase change materials based on encapsulated metal and alloy micro- and nano-particles with advanced thermophysical properties for cyclable and stable thermal energy storage/release is highlighted.

## Graphical abstract

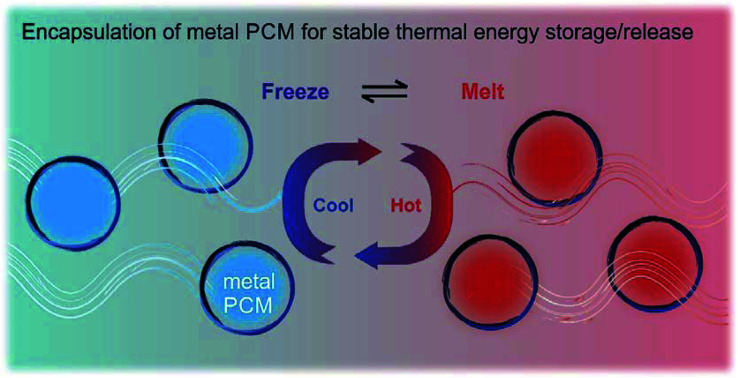

## 1. Thermal energy storage (TES)

When we look back at the history of humankind, the discovery of fire helped our ancestors move from the life of savages into the most dominant species on earth and in creating fantastic civilisations. Using fire for cooking is one of the earliest applications of thermal energy, in which chemical energy contained in wood is transformed into thermal energy by combustion and then transferred to food *via* the heating process, ultimately resulting in physical and chemical changes in the food being cooked.

By April 2019, the human population on the Earth was ∼7.7 billion. Such large population numbers entail ever-increasing demands for different types of energy, including electricity, heat, and mechanical work. According to the International Energy Agency (IEA), world power generation in 2016 was 24 973 TWh, of which power from coal and natural gas accounted for 38.4% and 23.2%, respectively.^1^ According to this data, energy generation is dominated by burning fossil fuels, which has resulted in massive emission of greenhouse gases like CO_2_, thus contributing to global warming. As a consequence of global warming, ice sheets in polar regions are melting at an increasing rate, potentially causing a global sea-level rise.^2^ Furthermore, due to the limited reserves and non-renewable characteristics of fossil fuels, we cannot depend completely on fossil fuels, as evidenced by the energy crisis that occurred in the 1970s.

### 1.1. Necessity of thermal energy storage and management

**Fig. 1:**
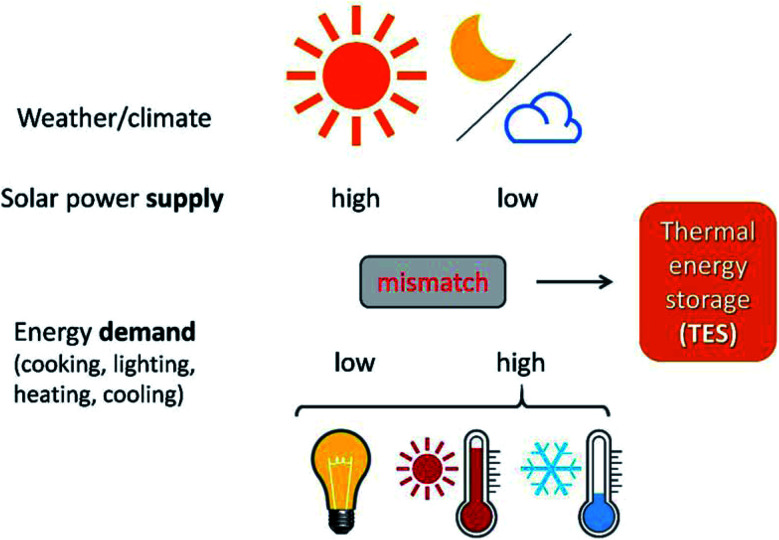
TES helps solve the time and space mismatch between the fluctuating solar energy supply and energy demand for human activity.

TES is crucial when attempting to harvest renewable energy resources *via* energy conversion and storage. Renewable energy resources refer to resources that can be ‘renewed’ in a human timescale. Solar energy, as one typical example of renewable energy, can be directly collected as thermal energy for utilisation in concentrated solar power plants (CSPs). However, the biggest problem with solar energy is related to its fluctuating availability, depending on time and space (Fig. 1). On the one hand, there is often a large mismatch between the peak hours of energy demand and consumption. For example, there is a big demand for air conditioning during summer nights in tropical areas, but no solar energy is available after sunset. On the other hand, due to geographical locations and climate, the distribution of solar energy varies across the planet (Fig. 1). For example, the solar resource available in the Sahara Desert is much larger than that in Hokkaido Island because of the differences in latitude; while the solar resource available in Yangtze Plain, located at the same latitude, is much less abundant than that in the Sahara Desert due to differences in the climate. Besides, the large-scale solar thermal power plants currently being used are set in remote areas, while the densest consumption of energy is from urban areas with a large population. This time and space mismatch of demand and supply in solar thermal energy for electricity production and supply can be addressed using TES.

High-grade thermal energy, if not consumed or stored, will simply dissipate into the environment as waste heat. This will result in irreversible degradation in thermal energy quality. In addition to CSP plants, a large amount of thermal energy is generated in other activities, such as industrial waste heat and heat from nuclear power plants. Therefore, TES is a good way for storing these energies for conversion to other useful forms, instead of simply dissipating into the environment.

In addition to storage, proper management of thermal energy, *i.e.*, heat dissipation from high-performance electronic devices, is an important consideration in modern society.^3^ Good thermal management of mobile phones can reduce damage to the central processing unit (CPU) and the batteries, increase battery life and improve the comfort of use. Since the development of the first mobile phone, it has gradually transformed into a more compact and lighter device. However, the constant developments in information and communications technology make it challenging to cool mobile phones sufficiently, especially in the new 5G (5th generation wireless systems) era. The changes in the cooling requirements of 5G mobile phones mainly come from two aspects, namely an increase in power consumption and changes in mobile-phone structure. In terms of power consumption, the 5G mobile phone possesses a more powerful processor and higher data-processing capability than a 4G phone. As a result, the absolute value of the produced heat increases sharply, which makes it difficult to dissipate heat. Besides, structural changes in mobile phones impose higher requirements on heat-dissipation performance. As the number of 5G antennae increases and the penetration of electromagnetic waves becomes weaker, phone manufacturers are slowly turning toward non-metallic bodies, which require an additional heat-dissipation design. Meanwhile, with the recent progress in chip manufacturing, more compact mobile phones are being designed, which also contributes to difficulties in thermal management. Since latent heat can be stored at a constant temperature in phase-change materials (PCMs), these materials can be used as heat sinks that can absorb heat produced from modern compact electronic devices.

### 1.2. Classification of TES methods

Based on the applications, TES can be achieved by sensible heat storage (SHS), latent heat storage (LHS), and chemical heat storage (CHS).^4^Table 1 summarises the principles of heat storage/release, the quantity of the stored energy, storage density, advantages, disadvantages, and requirements of the materials for each method. Briefly, SHS can allow fast charge/discharge by increasing or reducing the temperature of storage materials (eqn (1), Table 1). SHS has heat loss and low volumetric TES density. In contrast, LHS realises high-density TES by absorbing energy at a constant temperature (eqn (2), Table 1) *via* phase changes of the TES materials, called PCMs. Hence, PCMs are promising for solar TES or as heat sinks for heat dissipation of mobile phones. CHS achieves high-density TES^5^ by breaking/making chemical bonds to store/release energy (eqn (3) and (4), Table 1).^6^ Their drawbacks relate to the chemical reactions involved, *e.g.*, MgH_2_/Mg CHS needs H_2_ storage and strict reaction conditions (*e.g.*, 50–100 bar).^6,7^

**Table 1:** Characteristics of typical TES methods

	Thermal energy storage		
	Sensible heat storage (SHS)	Latent heat storage (LHS)	Chemical heat storage (CHS)
Principle of heat storage/release	Temperature increase/decrease in storage materials (w/o phase change)	Phase change of storage materials	Forming/breaking bonds in chemical reactions
Example	Waters, rock, concrete	Paraffin, molten salts (*e.g.*, NaNO_3_), eutectics	MgH_2_(s) + Δ*H*_r_ ↔ Mg(s) + H_2_(g) (3)
Stored/released energy, *Q* (J)	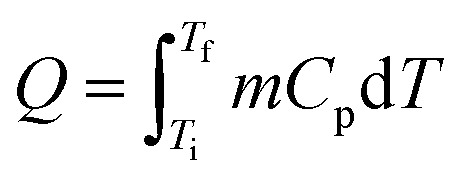 (1)	*Q* = *m*Δ*H* (2)	*Q* = Δ*H*_r_ (4)
	*m* (kg): mass of materials, *T*_i_ (K): initial temperature *T*_f_ (K): final temperature, *C*_p_ (J kg^−1^ K^−1^): specific heat capacity, Δ*H* (J kg^−1^): phase change enthalpy, Δ*H*_r_ (J kg^−1^): heat of reaction		
Storage density (J m^−3^)	Low	Moderate	High
Advantage	- Fast charge/discharge	- Store/release heat at a constant temperature	- Store at constant temperature possible
	- Reliable	- Large range of available *T*_m_	- Durability
Disadvantage	- Heat loss, short time storage	- Long time storage	- Long time storage, transportation
	- Need a large volume of materials	- Slow charge/discharge	- Strict reaction control
	- Temperature change during charge/discharge	- Supercooling	- Possibly involves in toxic substances
		- Some have low thermal conductivities	
		- High cost	
Requirement of storage materials	High heat capacity	- High latent heat	- Suitable enthalpy
		- Right phase change temperature	- Reversible and no side reaction
	Stable, non-toxic, low cost, feasible for transport and storage		

**Fig. 2:**
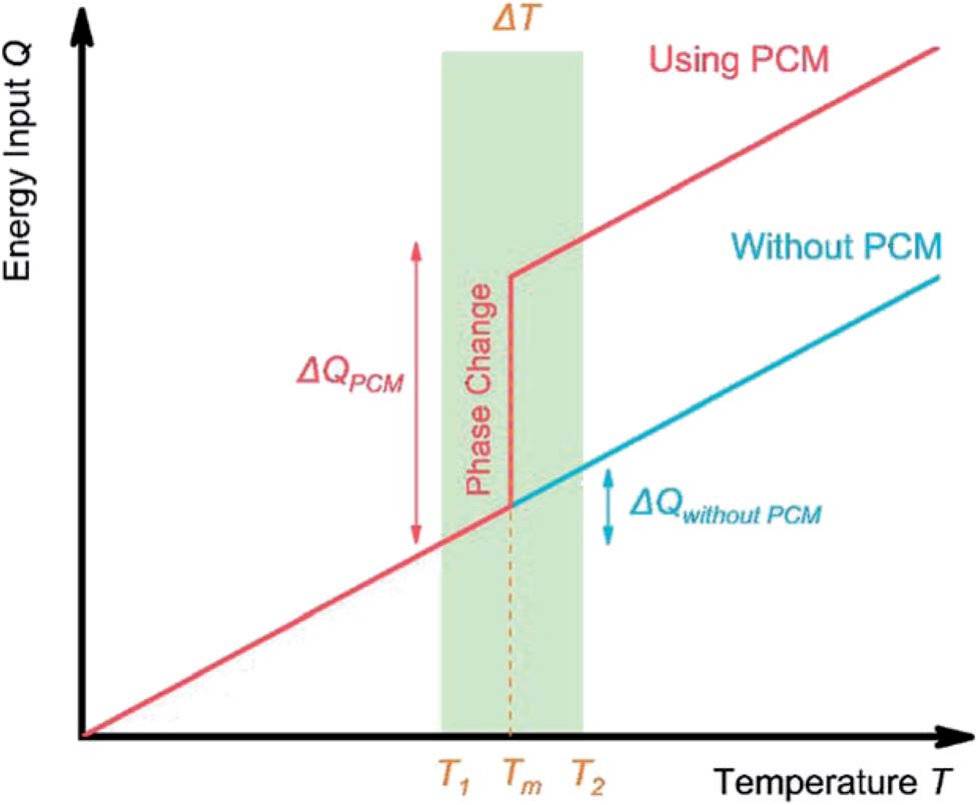
TES process in a solid–liquid PCM. Near the melting point *T*_m_ of PCM, the TES system containing PCM has a higher TES capacity compared with a system without PCM.

Thermal energy stored in PCMs, *Q* (J), in a certain temperature range from *T*_i_ to *T*_f_ comprises sensible heat from the increase in temperature and latent heat from the phase change process (eqn (5)). In a narrow range of temperatures near the melting point (green area, Fig. 2), the TES capacity of PCMs is much higher than that of SHS.5
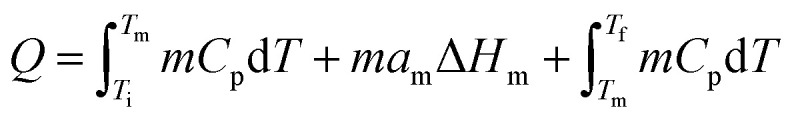
where *T*_m_ is the melting temperature (°C), *a*_m_ is the mass fraction of the melted PCM, and Δ*H*_m_ is the melting enthalpy (J kg^−1^).

## 2. Phase-change materials

### 2.1. PCM selection criteria

**Fig. 3:**
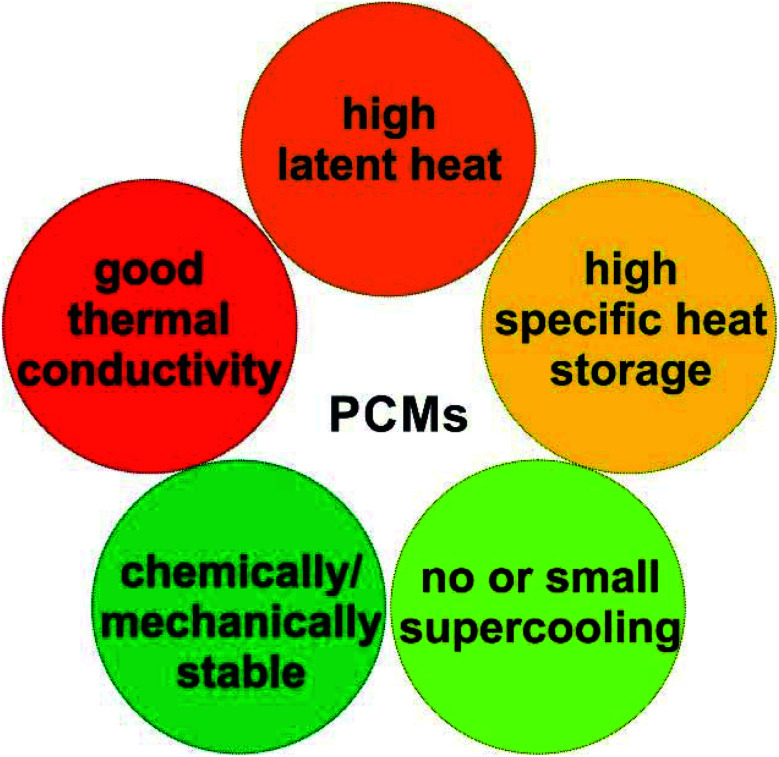
Basic selection criteria of PCMs.

An ideal PCM for TES should possess the desired properties (Fig. 3) to meet the demands of a specific application.^8^ Below the main requirements are discussed in brief.

#### 2.1.1. Thermal properties

PCMs should exhibit high latent heat per unit volume or weight depending on the application. A high LHS volumetric density is important for compact storage units to reduce the costs of steady TES systems (CSP, waste heat recovery). In lightweight systems, PCMs of a high TES gravimetric density are required. Further, the phase change temperature should match the desired operating temperature in the TES system. During melting, the change in the molar entropy of the system (Δ*S*) is positive. Hence, enthalpy of melting (Δ*H*_m_) given in eqn (6), thereby, LHS, increases with the melting temperature (*T*_m_).6
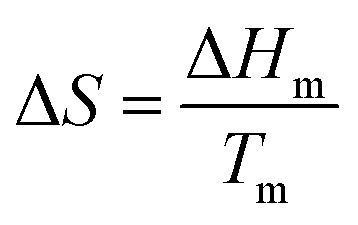


Meanwhile, PCMs with high specific heat are expected to provide additional SHS in the same temperature range. Importantly, a high thermal conductivity is desired for rapid heat storage and release and reduces the area of the heat-exchange surface.

#### 2.1.2. Physical properties

Volume changes of PCMs during the phase-change process should be minimised not to damage the PCM container and heat exchanger. Meanwhile, to avoid evaporation and deterioration, a PCM should exhibit low vapor pressure at the operating temperature. Reversible phase change and congruent melting are preferred for stable cycling. Besides, PCMs of high density may exhibit higher volumetric TES density.

#### 2.1.3. Kinetic properties

The supercooling effect of a PCM should be as small as possible. When supercooled, a liquid does not freeze into a solid even when the temperature is reduced to below its freezing point. This delays heat release, lowering TES efficiency. Instead, fast crystallisation is preferred to increase the dynamic response of the entire TES system.^8^

#### 2.1.4 Chemical properties

The selected PCM should be non-toxic, non-flammable, non-explosive, and non-corrosive to the metal containers. When cycling, there should be no chemical decomposition or side reactions to ensure the long-term stability of the system.

### 2.2. Classification of PCMs

PCMs are classified into solid–solid, solid–liquid, liquid–liquid, liquid–gas, and solid–gas PCMs based on the physical state of PCMs before and after phase transition. For TES, liquid–gas and solid–gas PCMs are not preferred as they entail drastic volume changes. Meanwhile, solid–solid PCMs^9,10^ generally show a small latent heat of phase transition though they exhibit minimal volume changes. Solid–liquid PCMs, hereafter simply referred to as ‘PCMs’, are the most studied and used media for TES. Table 2 lists the main types, examples and properties of solid–liquid PCMs.^11–29^

**Table 2:** Solid–liquid PCMs, example materials and their properties^11–29^^a^

	Sub-category	Example of materials	*T*_m_ °C	*C*_p_ kJ kg^−1^ K^−1^	*ρ* kg m^−3^	*λ* W m^−1^ K^−1^	Δ*H*_m_ kJ kg^−1^	Ref.
Organics	Paraffin	*n*-C_19_H_40_	32	2.27_l_	785	0.213–0.152	222	11–13
		*n*-C_30_H_62_	65.4	1.30	775	0.571–0.153	252	11–14
	Fatty acid	*n*-C_10_H_20_O_2_	32.0 ± 1.5		850 ± 80	0.15	145 ± 15	15 and 16
		*n*-C_18_H_36_O_2_	68.4 ± 1.5	2.830	840 ± 80	0.30	211 ± 21	15 and 17
	Ester	*n*-C_18_H_35_O_2_CH_3_	37.0	1.75 × 10^−3^	852		160.7	18 and 19
	Polymer	PEG-400	5.8 ± 0.8		1126	0.185	84.1 ± 7.7	20 and 21
		PEG-6000	62.1 ± 0.6			0.187 ± 0.002_s_	180.1 ± 4.5	21
Inorganics	Salt hydrate	CaCl_2_·6H_2_O	24	1.4_s_	1470	1.09_s_	140	22
				2.1_l_		0.54_l_		
	Moten salt	NaNO_3_	309.3	1.798–1.812	NA	0.514	174.13	23–25
	Eutectic salt	53% NaNO_3_, 40% NaNO_2_, 7% NaNO_3_ (wt%)	142	1.40_s_	1790	0.7	∼65–67	26 and 27
				1.56_l_				
	Metal	Ga	29.8	0.37	5907	29.4_l_	80.12	28
	Alloy	Bi_58_Sn_42_	138	0.201	8560	19_l_	44.8	28
		Sn–Bi–Pb–Zn	90.78–171.75	0.137–0.277	7720–10 110	9.54–41.76	19.59–59.8	29

a *T*_m_: melting point, *C*_p_: specific heat capacity, *ρ*: density, *λ*: thermal conductivity, Δ*H*_m_: melting enthalpy, “l” and “s” (lower case) are “liquid” and “solid” state, respectively.

#### 2.2.1. Organic compounds as PCMs

Organic PCMs have been widely studied for low-temperature TES applications based on their low melting points (<200 °C).^30^ Paraffins, fatty acids, esters, and polymers (*e.g.*, polyethylene glycol, PEG) exhibit high LHS capacities (Table 2). However, their drawback is low thermal conductivity (<1.0 W m^−1^ K^−1^), which reduces the TES charge–discharge rate (Table 2). The melting point of each type of organic PCMs increases with an increase in their molecular weight (Table 2). Thus, by varying the number of C chain or monomer units, the phase change temperature of organic PCMs can be well-tailored. When the molecular weight increases, the crystallisation degree of fatty acids and the crystallinity of PEG increases, resulting in higher melting enthalpies. Fatty acids show little supercooling and small volume changes upon phase transition. Fatty acid esters can form eutectic mixtures for little or no supercooling. In TES applications, fatty acids have an unpleasant smell and are more expensive than paraffins. The former corrodes metals whereas the latter does not.

#### 2.2.2. Inorganic PCMs

Inorganic PCMs provide a wide range of operating temperatures in both low- and high-temperature LHS applications (Table 2). The materials most often studied as PCMs include salt hydrates,^31–35^ molten salts (and their eutectics),^36–40^ metals, and alloys.^41–55^

Salt hydrates are inorganic salts containing crystallised water, such as calcium chloride hexahydrate (CaCl_2_·6H_2_O). Their solid–liquid phase-change process is accompanied by salt dehydration and hydration.^11,33^ However, a major chunk of the currently available salt hydrates undergo incongruent melting, that is, the dehydrated salts cannot dissolve in water at the melting temperature, causing sedimentation and phase separation. Besides, their poor nucleation ability causes supercooling.^11,34,35^

Molten salts can also function as PCMs for low to high-temperature TES (∼100 to 1000 °C).^36^ They have a high volume expansion ratio during phase transition, and can corrode metallic containers.^38–40^ Although salt hydrates and molten salts have low thermal conductivity (below ∼1.0 W m^−1^ K^−1^), they possess high TES density, acceptable price, and abundant reserve for being applied in TES.^31–33,37–40^

Metal and alloy PCMs with high melting temperatures (>300 °C) are usually used as PCMs in high-temperature LHS systems. Metals and alloys that can melt below 300 °C are referred to as low melting-point metals and used for low-temperature PCMs. For TES, their superior thermal conductivity (∼1.0 W m^−1^ K^−1^, Table 2) is advantageous, as it eliminates the need for large heat-exchange surfaces. Moreover, metallic PCMs have high density and larger TES volumetric densities than other types of PCMs, good cycling stability^41,42^ and low volume expansion.

Examples of high-temperature metallic PCMs include binary and ternary alloys containing Al, Cu, Mg, and Zn, such as Al–Si, Al–Si–Mg, Al–Si–Cu, and Al–Mg–Zn alloys. They exhibit the highest enthalpy of fusion at a given volume.^42–44^ High-temperature metallic PCMs have been proposed for application in solar power-generation systems.^45,46^ While several low-melting-point metals such as Bi, Ga, Sn, In, Zn, Cd, Te, Sb, Tl, Hg, and Pb, can function as PCMs, Sn, Bi, In, and Ga are often preferred due to their low toxicity and good physical and chemical properties. The physiothermal properties of various low melting-point metals and alloys are listed in Table 3.^28,29,47–49^

**Table 3:** Physical properties of low melting point metals and alloys^a^

Metals and alloys	*T*_m_ °C	*T*_b_ °C	*C*_p,l_ kJ kg^−1^ K^−1^	*ρ* kg m^−3^	*λ*_l_ W m^−1^ K^−1^	Δ*H*_m_ kJ kg^−1^	TES density MJ m^−3^	Ref.
Ga_73.5_In_15.4_Sn_11.1_	10.6					69.03		47
Ga_78.4_In_14.9_Sn_6.7_	10.9					71.2		47
Ga_83.5_In_16.5_	15					71.68		47
Ga_91.6_Sn_8.4_	19.8					78.29		47
Ga_95_Sn_5_	19.9					79.22		47
Ga_97.9_Al_2.1_	26.5					82.59		47
Cs	28.7	2023.8	0.236	1796	17.4	16.4	29.45	28
Ga	29.8	2204.8	0.37	5907	29.4	80.12	473.27	28
Rb	38.9	685.73	0.363	1470	29.3	25.74	37.84	28
Bi_44.7_Pb_22.6_In_19.1_Sn_8.3_Cd_5.3_	47		0.197	9160	15	36.8	337.09	28
Bi_35.5_In_64.5_	54.1					30.82		47
Bi_49_In_21_Pb_18_Sn_12_	58		0.201	9010	10	28.9	260.39	28
Cerrolow	58					90.9		48
Bi_38.7_Sn_16.7_Pb_14.4_In_30.2_	58.3					28.98		47
Bi_32_In_51.2_Sn_16.8_	60.8					25.4		48
Bi–Cd–In eutectic	61					25		48
Bi_41.8_In_58.2_	61.4					29.88		48
K	63.2	756.5	0.78	664	54	59.59	39.57	28
Bi_50_Pb_26.7_Sn_13.3_Cd_10_	70		0.184	9580	18	39.8	381.28	28
Cerrobend	70					32.6		48
Bi–Pb–In eutectic	70					29		48
Bi_41.6_Sn_19.4_Pb_23.2_Cd_15.8_	71.7					24.51		47
Bi–In eutectic	72					25		48
Bi_21.8_In_78.2_	73.1					22.46		47
Bi_38.5_Sn_22.2_Pb_25.3_In_14_	75					25.36		47
Bi_40.5_Sn_28.5_Pb_16.3_In_14.7_	75.7					21.71		47
Bi_53.8_In_27_Sn_19.2_	76.6					32.6		49
Bi_42.5_In_35.2_Sn_22.3_	79.2					36.91		47
Sn_9.5_Bi_56_Pb_34.5_	90.8		0.161			19.54		29
Bi_71.2_Pb_28.8_	94.9					28.99		47
Bi_52_Pb_30_Sn_18_	96		0.167	9600	24	34.7	333.12	28
Bi–Pb–Sn eutectic	96							49
Na	97.8	881.4	1.38	926.9	86.9	113.2	104.95	28
Bi–Pb	125							49
Bi_55_Pb_43_Zn_2_	127		0.154			20.44		29
Sn_48_Bi_50_Zn_2_	135		0.198			47.62		29
Bi_58_Sn_42_	138		0.201	8560	19	44.8	383.49	28
In	157	2023.8	0.23	7030	36.4	28.59	200.99	28
Sn_73.5_Pb_22_Zn_4.5_	172		0.247			59.8		29
Li	186	1342.3	4.389	515	41.3	433.8	223.4	28
Sn_91_Zn_9_	199		0.272	7270	61	32.5	236.28	28
Sn	232	2622.8	0.221	730	59.6	60.5	44.17	28
Bi	271	1560	0.122	979	8.1	53.3	52.18	28

a *T*_m_: melting temperature; *T*_b_: boiling temperature; *C*_p,l_: specific heat capacity (in liquid); *ρ*: density; *λ*_l_: thermal conductivity (liquid); Δ*H*_m_: melting enthalpy.

Sn can be applied in solar power plants for TES. Lai *et al.* demonstrated enhanced solar-thermal storage by exploiting the latent heat of Sn/SiO_*x*_ core–shell nanoparticles (NPs) embedded in HITEC salt, a eutectic mixture of 53 wt% KNO_3_, 40 wt% NaNO_2_ and 7 wt% NaNO_3_. The heat capacity of HITEC containing Sn/SiO_*x*_ was increased by 30% compared to that of HITEC solar salt.^50^

Low-melting-point metal PCMs can be used in thermal comfort applications. For instance, the melting temperature of Ga is ∼29.8 °C, which is close to room and body temperatures. Its TES volumetric density (∼473.27 MJ m^−3^) is much larger than that of a paraffin material with a similar phase-change temperature, *n*-nonadecane (174.27 MJ m^−3^; 32.0 °C).^51^ More importantly, Ga exhibits a large thermal conductivity of 29.28 W m^−1^ K^−1^ in the liquid state, which enables temperature control. Moreover, it is relatively non-toxic, non-flammable, non-explosive, and shows stable cyclic energy storage and release characteristics with small volume expansion.

Low-melting-point metal PCMs have been investigated for heat dissipation in electronic devices. Ge *et al.* used Ga to keep a smartphone cool during operation and demonstrated that 3.4 mL of Ga maintains the module at a temperature below 45 °C for 16 min at 2.832 W. This holding time for maintaining operational temperature was longer than that of most conventional organic PCMs.^52^ Yang *et al.* developed a finned heat pipe-assisted passive heat sink based on low-melting-point metal PCMs for buffering thermal shock with a heat generation rate of up to 1000 W (10 W cm^−2^); this resulted in longer operation times (1.4–2.4 times) in high-power electronics when compared to cases in which conventional organic PCMs were used.^53^

However, there is still much scope for improving metals and alloys as high-performance PCMs for the storage and management of thermal energy. For example, the corrosion of metal PCMs and the sintering of liquid–metal particles may lead to changes in their structure or morphology of PCMs during operation, inhibiting their use in the form of nanofluids or slurries.^54,55^ Therefore, the encapsulation of metal PCMs in macro-, micro-, or nano-scale vessels should be considered to improve their utility.

## 3. Micro/nano encapsulation of PCMs

Compared to their bulk counterparts, PCMs in micro- or nano-size exhibit much larger surface areas. For example, as for two samples (same weight and density) of spherical particles with two different diameters: 1 cm and 100 nm, the surface area of the latter sample is 100 000 times larger than that of the former one. Hence, PCMs of micro- or nano-size particles can allow for faster heat transfer through their large surface area during charge/discharge between PCMs and the matrix. The encapsulation is important to protect the high surface area of micro- or nano-PCMs from sintering. Further, the surface modification of micro/nano-size PCMs is easy to achieve and they can be dispersed in a heat transfer fluid (HTF), which enhances the application potential of PCMs.

**Fig. 4:**
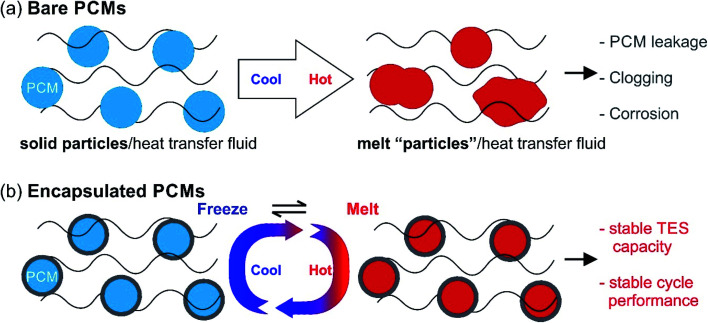
(a) Bare PCMs in HTF during melt-freeze cycle test undergo aggregation, sintering, leakage, *etc.*, causing clogging and corrosion to the container. (b) The problem can be solved by encapsulating the PCMs for cycle stability in HTFs.

Despite the suitability of PCMs in many thermal applications, the practical use of solid–liquid PCMs is still limited due to several issues. Liquid PCMs might leak during the heat-storage process (Fig. 4a), which decreases their TES capacity and cyclic stability. In particular, when using acids and molten salts as PCMs, corrosion may occur on key parts of the devices or facilities, reducing their service life or even causing safety problems (Fig. 4a).

One solution to these issues is to encapsulate PCMs at the micro- or nano-scale (Fig. 4b). The encapsulated PCMs are called micro-encapsulated PCMs (MEPCMs) or nano-encapsulated PCMs (NEPCMs), depending on PCM dimensions (MEPCMs: 0.1–1000 μm; NEPCMs: 1–100 nm). Encapsulation refers to the process of embedding PCM cores (can be single-core or multi-core) into a shell or layer (protection structure). This shell thus isolates and protects PCM cores from the external environment. By micro/nano encapsulation, PCMs can be transformed into powder or paste form. By using the porous shell structure the volume expansion of PCMs can be no longer an issue. Several reports have been published on the synthesis of MEPCMs and NEPCMs.^56–60^ The materials used for encapsulation (encapsulant) vary from organics (such as polystyrene, urea formaldehyde, and polymethyl methacrylate) to inorganic materials (such as calcium carbonate, silica, sodium silicate, and metal oxides).^61^ The typical preparation methods of MEPCMs and NEPCMs are discussed in the rest of this section.^62^

### 3.1. Approaches for micro/nano encapsulation of PCMs

The methods available for PCM micro/nano encapsulation can be classified into three main categories, *viz.* polymerisation methods (sometimes also named as ‘chemical methods’), physico-chemical methods, and physical methods.

#### 3.1.1. Polymerisation

##### Emulsion polymerisation

In emulsion polymerisation, one phase containing the monomer is uniformly dispersed in the continuous phase using a surfactant and an emulsifier *via* mechanical stirring to form an emulsion. The encapsulating shell is then grown on the surface of the PCM core.^63,64^ The emulsion droplet size is in the range of 1–10 mm and can be further decreased to 20–200 nm by providing higher external energy. Stable isotropic liquids with two or more separated phases in equilibrium are also called microemulsions. Meanwhile, nanoemulsions (droplet size < 100 nm) can be formed by adding a large amount of surfactant to overcome the interfacial energy. While the size of the droplet does not provide a clear distinction between nano- and micro-emulsions, their thermodynamic stability does. Compared to microemulsions, nanoemulsions are thermodynamically unstable.^63^

##### Interfacial polymerisation

In interfacial polymerisation, the first reactive monomer in one phase is dispersed in an immiscible phase containing the second monomer. The monomers react at the interface to form a polymeric membrane that encapsulates PCMs.^65^ Usually, interfacial polymerisation yields MEPCMs.^66^

##### *In situ* polymerisation

In *in situ* polymerisation, two immiscible phases containing organic intermediates react with each other to yield the encapsulant.^67^ Unlike in the case of interfacial polymerisation, there are no reactants in the core material during *in situ* polymerisation and all polymerisation reactions occur in the continuous phase.^59^ Usually, *in situ* polymerisation yields particles in the size range of 5–100 μm.^67^

#### 3.1.2. Physico-chemical methods

##### Coacervation

In the coacervation process, liquid–liquid phase separation (bottom: polymer-rich dense phase; top: transparent solution) is induced in a homogeneous solution of charged macromolecules by the addition of natural salt or alcohol.^68–73^

##### Sol–gel encapsulation

The sol–gel method of PCM encapsulation often starts with a suspension of PCM nanoparticles or microparticles in an aqueous solution. The formation of the encapsulating shell (hydrated metal or semi-metal oxide, such as TiO_2_ and SiO_2_) is induced by hydrolysing shell precursors to produce soluble hydroxylated monomers, followed by polymerisation and phase separation to form the encapsulating shell (for example, SiO_2_ and TiO_2_).^74^

##### Self-assembly method

The self-assembly method is sometimes also called as the ‘one-step method’. When this method is used to encapsulate PCMs, system components such as molecules, polymers, colloids, or macroscopic particles are organised into shells or other confinement structures by promoting local interactions among these components, without external direction.^75–77^

##### Solvent evaporation

PCM encapsulation by solvent evaporation generally consists of four steps: (1) dissolution of hydrophobic PCM in an organic solvent containing the polymer to be coated, (2) emulsification of the organic phase containing the PCM in a continuous aqueous phase, (3) solvent extraction from the organic phase containing the PCM by evaporation and transforming the droplets into solid PCM particles with polymer encapsulation, and (4) removal of residual solvent.^61,78^

##### Supercritical CO_2_-assisted methods

The easily accessible supercritical conditions of CO_2_, when CO_2_ stays as a liquid at or above its critical temperature and pressure, are 73.8 bar and 31.1 °C, which is close to the ambient temperature. Supercritical CO_2_, which is non-toxic, non-flammable, exhibits gas-like viscosity and liquid-like density, and is readily soluble, can be used as a solvent, antisolvent, solute, drying medium, and foaming agent.^66,79^

#### 3.1.3. Physical methods

Physical methods are often used for PCM encapsulation in large-scale industrial production. Although these techniques are not capable of producing microcapsules smaller than 100 μm because of their inherent characteristics, they are still suitable for the mass production of MEPCMs.^66^ Typical physical techniques include spray drying, fluid-bed coating, and electrostatic encapsulation.^61,80–82^

Among three methods for PCM encapsulation, polymerisation can provide uniform coating (*in situ* polymerisation), which is versatile in particle size and cost effective with good size control for PCMs.^66^ The method has been applied for organic PCMs, which results in particle size of 1–4000 μm. Physio-chemical method can also allow for good size control (sol–gel encapsulation, coacervation), but it is more suitable for small scale synthesis. The aggregation after coating should be considered. Sol–gel encapsulation is often used for inorganic coating of metal and alloy PCMs. The method can be used for PCMs of nanoscale size (<100 nm) and form uniform shell. The physical method is advantageous in terms of scaling up, simplicity, and low-cost for PCMs.^66^ The method, however, is not able to produce thin coating layer in nanoscale.

### 3.2. Micro/nano encapsulation of metals and alloys

A large number of studies have concentrated on the micro- and nano-encapsulation of organic compounds as conventional PCMs. In contrast, there are only a few studies on the micro- or nano-encapsulation of low-melting-point and high-melting-point metals/alloys. This section is devoted to reviewing these studies.

**Fig. 5:**
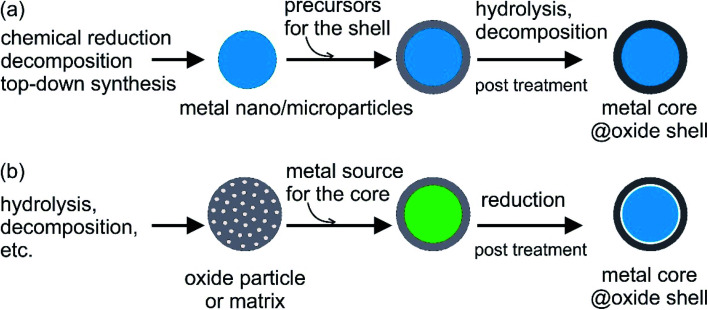
General approaches for oxide encapsulation of metal PCMs: (a) oxide coating on metal core and (b) reduction of metal source in the preformed oxide shell.

Metal oxides such as SiO_2_ and Al_2_O_3_ are among the most commonly used materials for coating PCMs. They are stable against oxidation, exhibit minimal corrosion, and are thermally stable over a large temperature range with relatively good thermal conductivity. There are two main approaches to synthesise encapsulated metal/alloy PCMs: (i) metal particles, which act as the cores, are prepared first, followed by coating with an oxide shell and (ii) reduction of the metal source distributed in the preformed oxide shell/matrix (Fig. 5). The metal cores used in the first approach can be synthesised by different methods and the core size can be tailored to yield different phase-change temperatures and heat capacities in different working environments (Fig. 5a). The dispersibility, reactivity in the solution used for coating (*e.g.*, water, alcohol, other organics, and biphasics), and surface properties of metal cores should be considered when choosing the coating method to ensure a complete coating without oxidation. This is especially important when coating is conducted on active metal nanoparticles, such as Sn and Bi. The hydrolysis of hydroxides in controlled pH conditions or thermal decomposition of hydroxide and/or nitrate is often used to generate oxide shells. Meanwhile, cores can be reduced in the presence of the shell using the second method. Cavities/voids can be expected in the resulting structure, which is beneficial for buffering volume changes and mechanical stress during the phase-change process in metal cores (Fig. 5b). In this case, the size and number of metal–core particles are not only controlled by the synthesis conditions but also by the oxide shell matrix.

#### 3.2.1. Micro/nano encapsulation of low-melting-point metals and alloys

##### Metal oxides or semi-metal oxides

In general, stable metal oxides or semi-metal oxides, such as SiO_2_, TiO_2_, and Al_2_O_3_, are often used to encapsulate low-melting-point metal nanoparticles and microparticles. Hong *et al.*^83^ used an emulsion method to prepare In nanoparticles by boiling commercial In powder in poly-α-olefin (PAO). The In nanoparticles thus produced were collected by centrifugation and encapsulated with silica (SiO_2_) using a sol–gel method. These SiO_2_-encapsulated nanoparticles were then suspended in PAO, which enhanced the heat-transfer properties of PAO for high-temperature applications. The SiO_2_ shell not only prevented the leakage and agglomeration of In in the liquid state, but also enhanced the dielectric properties of In nanoparticles, thus making them suitable for the cooling of electronic devices. The viscosity of PAO-containing SiO_2_-encapsulated In nanoparticles at 45 °C (9.49 cP) is close to the viscosity of PAO (4.68 cP). Therefore, the heat-transfer coefficient of PAO containing 30 at% In nanoparticles is 1.6 times higher than that of the original PAO.

**Fig. 6:**
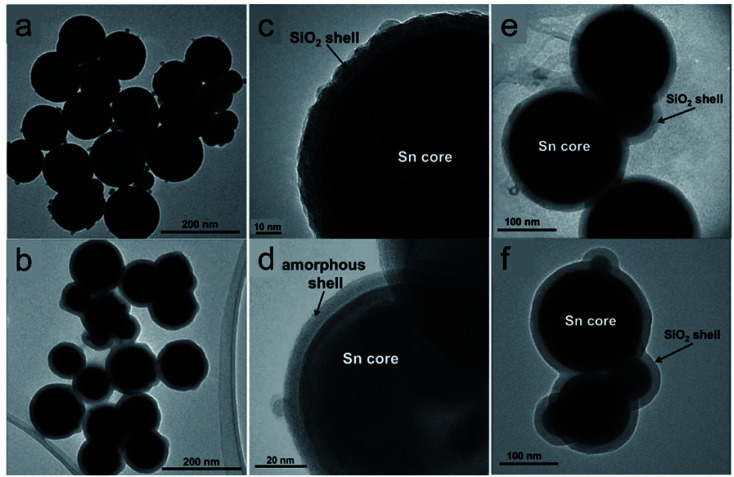
Silica encapsulated Sn nanoparticles (Sn@SiO_2_) for heat transfer and TES.^84^ TEM images of (a) as-prepared Sn and (b) Sn@SiO_2_ nanoparticles; high-resolution TEM images of (c) Sn@SiO_2_ and (d) Sn nanoparticles. TEM images of encapsulated Sn@SiO_2_ nanoparticles (e) before and (f) after 20 heating and cooling cycles. Reproduced from ref. 84 with permission from Wiley, USA, Copyright 2013 John Wiley & Sons, Ltd.

Cingarapu *et al.* synthesised Sn nanoparticles (Fig. 6a) using a modified polyol reduction method followed by sol–gel SiO_2_ encapsulation on the surface (Sn@SiO_2_) (Fig. 6b).^84^ The high-resolution TEM image of the encapsulated Sn@SiO_2_ nanoparticles (Fig. 6c) shows crystalline silica shell with a thick grain boundary between the shell and the Sn core whereas the unencapsulated Sn nanoparticles have an amorphous SnO_*x*_ layer formed on the surface (Fig. 6d). The SiO_2_ shell ensures the chemical and structural stability of Sn nanoparticles during melt-freeze cycling (Fig. 6e and f). By dispersing Sn@SiO_2_ core–shell phase-change nanoparticles (5 vol%) in a synthetic HTF, therminol 66 (TH66), enhanced thermal properties could be achieved. As for the thermal conductivity of the nanofluid, it increased by ∼13%, which agrees with Maxwell's effective medium theory. The volumetric TES of the nanofluid increased by ∼11% by cycling in the temperature range of 100–270 °C. This increase was attributed to the latent heat generated by the melting of Sn cores; this value might be further increased if thermal cycling is conducted in a narrower temperature range. In the range of 25–125 °C, the viscosities of the modified nanofluids and base fluid were quite similar. In addition, no changes were observed in Sn@SiO_2_ (5 vol%) during 20 heat-cool cycles, indicating the good thermal stability of this system. These experimental results thus illustrate the beneficial effects of Sn nanoparticles on both the thermal conductivity and TES density of base HTFs and highlight their potential for use in CSP systems.

**Fig. 7:**
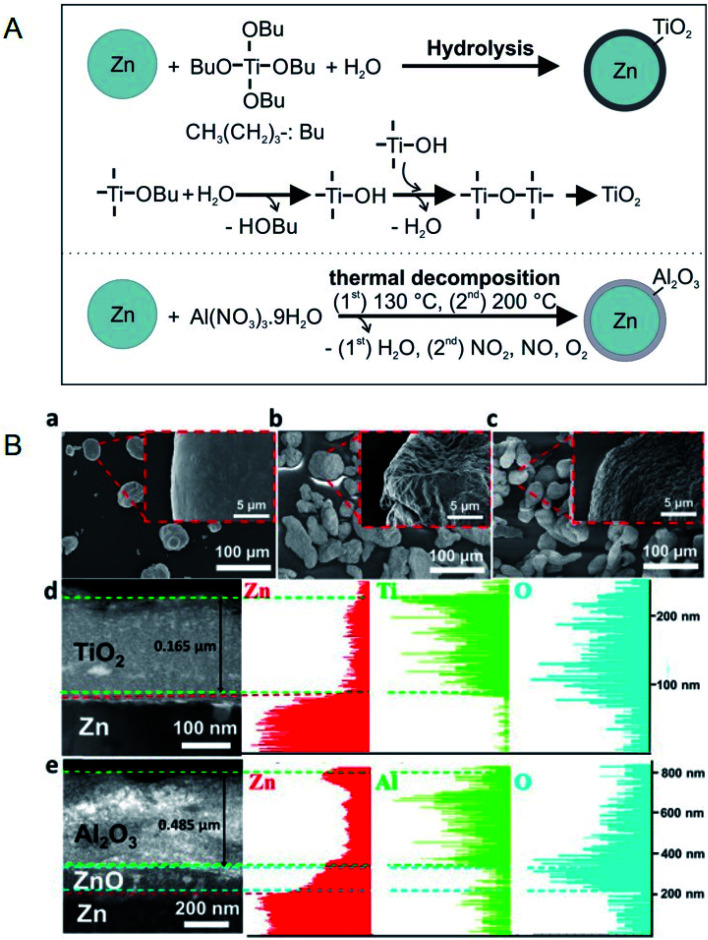
(A) Synthesis method for Zn/TiO_2_ microparticles and Zn/Al_2_O_3_ microparticles. (B) SEM images of (a) pure Zn, (b) Zn/TiO_2_, and (c) Zn/Al_2_O_3_ microparticles. (d and e) TEM cross-sectional images and EDS analysis for Zn/TiO_2_ and Zn/Al_2_O_3_ microparticles, respectively. Reproduced from ref. 85 with permission of Elsevier, Copyright 2018.

Hsu *et al.* prepared Zn@TiO_2_, Zn@Al_2_O_3_, and Zn@SiO_2_ core–shell microparticles by hydrolysis (Zn@TiO_2_ and Zn@SiO_2_) and thermal decomposition (Zn@Al_2_O_3_), as shown in Fig. 7.^85^ Zn–Sn@SiO_2_ core–shell microparticles by hydrolysis (for SiO_2_ coating) and thermal annealing (for Zn–Sn alloying).^86^ Zn@Al_2_O_3_ microparticles were dispersed in HITEC salt,^87^ which is designed as a HTF in CSP plants.^85^ The heat capacity of the salt could be enhanced by 6.7% by doping with 10 wt% Zn@Al_2_O_3_ microparticles, while its viscosity increased from 1.3 to 3 cP in the temperature range of 350–550 °C.^85^ The authors defined thermal hysteresis (TH) in phase-change core–shell microparticles as the difference between the melting and crystallisation temperatures and it increased with an increase in shell thickness and heat-ramping rate. A discussion on TH is important because the latent heat of PCMs cannot be released and restored during cycling if the TH is beyond the operating temperature range of a TES system. Moreover, among Zn@TiO_2_, Zn@Al_2_O_3_, and Zn@SiO_2_, Zn@Al_2_O_3_ exhibited the smallest TH owing to the higher thermal conductivity of Al_2_O_3_ when compared to TiO_2_ and SiO_2_.

**Fig. 8:**
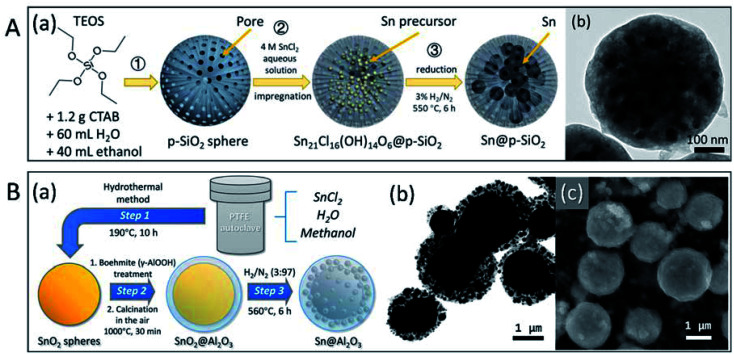
Micro and nano encapsulated Sn-based PCM particles. (A) Sn NPs@p-SiO_2_: (a) 3-step synthesis method, (b) TEM image; (B) size-tunable Sn@Al_2_O_3_: (a) 3-step synthesis method, (b) TEM and (c) SEM images of Sn@Al_2_O_3_. Reproduced from ref. 88 and 89 with permission of The American Chemical Society, Copyright 2018 and 2019, respectively.

Because low-melting-point metal particles, especially nanoparticles, are easily oxidised during synthesis and encapsulation, the direct encapsulation of pre-synthesised microparticles and nanoparticles may cause serious oxidation. Moreover, the synthesis and coating of metal nanoparticles involves complex procedures, which are not suitable for large-scale production. Previously, our group presented an encapsulation strategy, in which formation of oxide shell or other treatments were conducted on oxide shells before the formation of metal microparticles and nanoparticles.^88,89^ By maintaining the designated form and avoiding the leakage or sintering of Sn-based PCMs at the nano- and micro-level, the isolation/protection of oxide structures effectively enhanced the thermal stability of PCMs in melt-freeze cyclic working conditions. SiO_2_ (ref. 88) and Al_2_O_3_ (ref. 89) were chosen for the stabilisation of low-melting-point metal nano- or micro-PCMs (Fig. 8). Sn nanoparticles encapsulated in porous SiO_2_ particles, Sn NPs@p-SiO_2_, were prepared by the hydrogen reduction of SnCl_2_-absorbed porous SiO_2_ (p-SiO_2_) spheres. This resulted in Sn nanoparticles (∼30 nm) uniformly distributed in spherical SiO_2_ (Fig. 8A). The porous SiO_2_ matrix effectively prevented the coalescence of Sn nanoparticles and maintained the morphology and melting characteristics of the system with negligible changes during freeze-melt cycling. Size-tunable Sn@Al_2_O_3_ PCM particles with a core–shell structure (Fig. 8B) were fabricated using a 3-step method, including hydrothermal synthesis of SnO_2_ particles, boehmite treatment and air calcination, and hydrogen reduction. The as-obtained Sn@Al_2_O_3_ showed a core–shell structure; metallic Sn core located at the centre was covered with an Al_2_O_3_ shell with small Sn nanoparticles distributed inside. These Sn@Al_2_O_3_ particles exhibited a high PCM content (92.37 wt%) and showed stable thermal behaviour and morphology during 100 melt-freeze cycles in air. Our results showed that both the SnO_2_ matrix and Al_2_O_3_ shell effectively enhanced the thermal storage stability of Sn-based PCMs, which is indicative of their excellent potential in applications involving high temperatures (where organic PCMs are not suitable).

##### Self-encapsulation

**Fig. 9:**
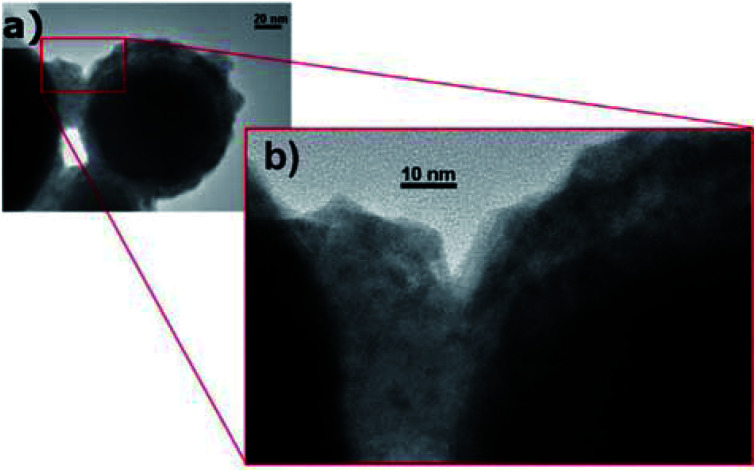
TEM images of self-encapsulated Sn@SnO_2_ particles. Reproduced from ref. 90. Published by Springer Nature. Copyright 2017.

To protect low-melting-point metal PCMs, self-encapsulation is another approach that may be employed. Navarrete *et al.* used Sn@SnO_*x*_ nanoparticles as self-encapsulated NEPCMs using the oxide shell that is formed on these entities when they are exposed to air (Fig. 9).^90^ When Sn@SnO_*x*_ was dispersed in commercial thermal oil (Therminol 66) to form nanofluids, the thermal conductivity and heat capacity of the base fluid increased. This was possible due to the high thermal conductivity of Sn and the latent heat generated by the melting of Sn nanoparticles. The thermal stability of Sn@SnO_*x*_ in both powder and nanofluid forms was verified by melt-freeze cyclic tests. Meanwhile, non-eutectic metal alloy (Sn/Pb) nanoparticles, in which solid and liquid phases coexisted during heating, exhibited heterogeneous crystallisation, which reduced their tendency to undergo supercooling when compared to completely melted nanoparticles.

##### Polymers

Polymer shells can also be applied for the microencapsulation of low-melting-point metals and alloys. Blaiszik *et al.* prepared a liquid metal alloy of Ga–In microencapsulated in urea-formaldehyde (UF) by *in situ* polymerisation.^91^ The obtained microcapsules exhibited a 73 nm-thick shell and an ellipsoidal shape with major and minor diameter aspect ratios ranging from 1.64 to 1.08 (major diameters in the range of 3–245 μm). The major and minor diameters decreased with an increase in the agitation rate (stirring speed) of the reaction mixture containing liquid metals, monomers, and copolymer, and the aspect ratio decreased to 1.08 with further increase in the shear rate.

##### Carbon encapsulation

**Fig. 10:**
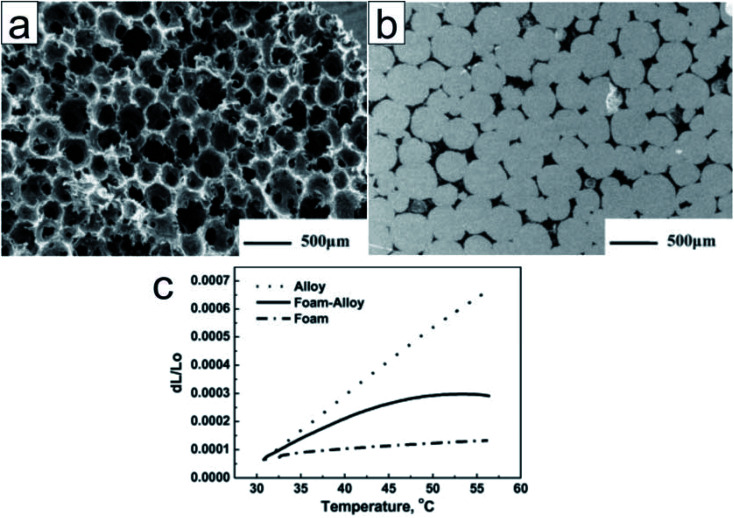
SEM image of (a) the unfilled graphite foam (b) the polished surface of the alloy filled graphite foam. (c) Thermal expansion of the Wood's alloy, graphite foam, and graphite foam/Wood's alloy composite. Reproduced from ref. 93 with permission of Elsevier, Copyright 2010.

Owing to their superior thermal conductivity, carbon materials are good candidates for encapsulating low-melting-point metals and alloys. Zhong *et al.* submerged graphite foams in a melted Wood's alloy (eutectic alloy of 50% Bi, 26.7% Pb, 13.3% Sn, and 10% Cd by weight)^92^ to form graphite foam/Wood's alloy composites (Fig. 10).^93^ The resultant composites exhibited a thermal conductivity (193.74 W m^−1^ K^−1^) twice as high as that of the alloy and graphite foam. In addition, the composite exhibited a significantly reduced thermal expansion coefficient (7.82 ppm K^−1^) when compared to the alloy (24.81 ppm K^−1^) (Fig. 10); however, there were no significant changes in latent heat. In addition, the composites exhibited enhanced mechanical properties (with the alloy in both solid and liquid phases) when compared to graphite foam.^93^ The researchers also impregnated compressed expanded natural graphite (CENG) with Wood's alloy; the thermal conductivity of the resultant composites was 2.8–5.8 times higher than that of Wood's alloy (58.88 W m^−1^ K^−1^). The latent heat of these composites (29.27–34.20 J g^−1^) makes them suitable for heat management in electronic devices.^94^

**Fig. 11:**
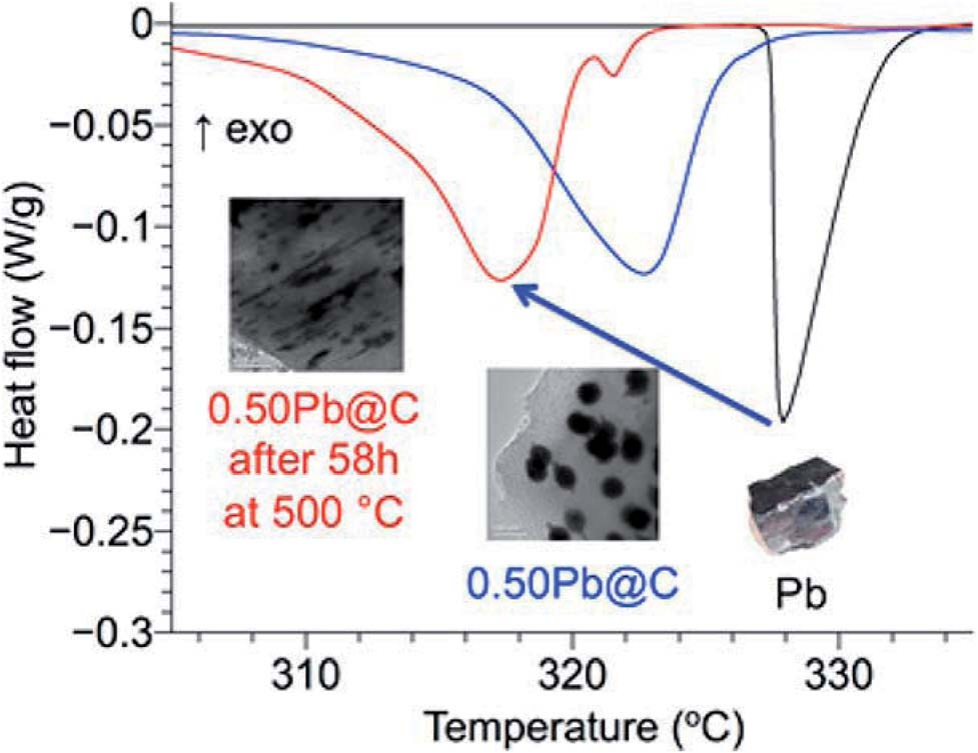
Pb nanoparticle–carbon matrix composites with tunable melting temperature as PCMs for TES. Reproduced from ref. 95 with permission of The American Chemical Society, Copyright 2018.

Tran *et al.* synthesised composites in which ligand-free metal nanoparticles (Bi or Pb) were distributed in a mesoporous carbon matrix to design form-stable PCMs (Fig. 11).^95^ The embedded metal nanoparticles helped in controlling the melting temperature of these composites (this was achieved by changing the size of the metal nanoparticles by tuning the amount of metal loaded inside the composites). A decrease in the melting temperature of both Bi and Pb nanoparticles in the composite materials compared to their bulk counterparts was observed. During 18 melt–freeze cycles, the phase-change temperature of the composites was found to be stable. Carbon, when used as an encapsulating material for metal nanoparticles, prevented the aggregation of metal nanoparticles, accommodated volume changes, and prevented leakage of liquid-state PCMs. In addition, porous channels in the carbon matrix served as containers for melted Pb nanoparticles.

After micro/nano encapsulation of low-melting-point metal PCMs, oxide shell thickness of tens to hundreds of nanometer has been observed.^83–86,88–90^ The observation of the surface of the core suggests that oxidation of the core occurred slightly during and possibly after the encapsulation.^83–86,88–90^ The decrease of melting enthalpy of the encapsulated PCMs over melt-freeze cycle in the air to a certain extent indicates additional oxidation occurred, especially, when the core size is below 100 nm,^88^ and the shell is not dense enough.^88,89^ Thicker and denser shells with bigger core sizes show less oxidation (or not report and better cycle performance (LHS)).^84–86,89^ Meanwhile, coating with oxide is advantageous for the cycle test in air. Having the metal PCMs in a carbon matrix, which has good thermal conductivity, allows for attaining encapsulated PCMs with high thermal conductivity.^76^ However, the method is reported for a core size of several hundreds of micron and it may be challenging for nano encapsulation. The summary of the particle size and thermos-physical properties of the encapsulated metal PCMs is given in Table 4. It can be seen that majority of the PCMs and encapsulating shells in the nanoscale are for low-melting-point metal PCMs. Studies for using the nano encapsulated PCMs in heat dissipation, TES or HTFs are expected to evaluate the performance of the metal PCMs for practical applications.

**Table 4:** Thermophysical properties of encapsulated metal PCMs^a^

Encapsulated metal PCMs	*d*_core_	*t*_shell_	*T*_m_ °C	*T*_c_ °C	*λ* W m^−1^ K^−1^	Δ*H*_m_ kJ kg^−1^	Cycle number	Ref.
In@SiO_2_ (68.8 wt% In) 30%/HTF-PAO	165–90 nm	10–80 nm	155.3	135		19.6	100	83
Sn@SiO_2_ (68.8 wt% In) 5%/HTF-therminol 66	50–100 nm	5 nm	228.3	98.3		48	20	84
			232					
Zn@TiO_2_	45.6 μm	160 nm				∼100	40	85
Zn@Al_2_O_3_ 1–20 wt%/HITEC salt	45.6 μm	450 nm				∼97	40	85
						+0.68–+13.6%		
Sn_x_Zn_1−*x*_@SiO_2_ 5%/HITEC salt	57.7 μm		278–429			67.41–96.09		86
Sn@SiO_2_	30 nm	400 nm*	229.1			8.46	100	88
						*6.62*		
Sn@Al_2_O_3_ (92.37 wt% Sn)	1–2 μm	100–700 nm	229.1	130.5; 142.7; 160.9		54.49	100	89
Sn@SnO_2_	103 nm	10 nm	225.5	106.6				90
	184 nm	10 nm	232.1	139.1				
Eutectic Sn–Pb@SnO_2_ 5%/HTF-therminol 66			184.14–284.48	175.64–173.76 (1^st^ peak)		27.48–44.62		90
In–Ga@urea-formaldehyde	245–3 μm							91
50Bi/27Pb/13Sn/10Cd@graphite foam					193.74	29.20		93
50Bi/27Pb/13Sn/10Cd@compressed expanded natural graphite					*λ*_*X*_ 164–344	29.27–34.20		94
					*λ*_*Y*_ 56–93			
Bi@C (0.25/1–2/1 w/w)	84–16 nm		265.4–238.8			5.04–16.90	18	95
Pb@C (0.5/1–1/1 w/w)	105–31 nm		322.8–310.1			5.67–2.42		95
Al–Cu (56.6 : 43.4 w/w)@Al_2_O_3_ (53 : 47 w/w) 0.5–10% NPs/(NaNO_3_–KNO_3_ 60/40 w/w)	85 nm	6–8 nm	546.74	540.95	0.480–0.335, 300 °C	∼146–161	150	96
						∼9–11 (10% NPs)		
Al–Si@α-Al_2_O_3_ (45 : 30 : 25 w/w)	40.7 μm	2.2 μm	573			247	10	58
			*573*			*245*		
Al-25 wt% Si@Al_2_O_3_	36.3 μm	1 μm	579	530		233	300	41
			*578*			*251*		
Al-30 wt% Si@Al_2_O_3_	44 μm		578			241	100	97
Al-20 wt% Si@Al_2_O_3_	29 μm		578			237		
Al-17 wt% Si@Al_2_O_3_	29 μm		578			272		
Al-12 wt% Si@Al_2_O_3_	37 μm		579			298		
Al–25Si@Al_2_O_3_	577 μm	6.9 μm (grain size)				183	3000	98
Al@α-Al_2_O_3_	36 μm		661–660			273–301	100	99
Al-12 wt% Si@Al_2_O_3_	100 μm		575.02			307.21		100
Al-12 wt% Si@Al_2_O_3_	100 μm		∼570–580			303.21	20	101
						*271.90*		
Al–Si@Al_2_O_3_						416.92–307.21		102
Al@Al_2_O_3_ (60–68 wt% core)	23.1 μm	1.4 μm	663.5	601.3–566.4		289–312	50	103
Al@Al_2_O_3_–C	20–50 μm		660.3	638.6	8	266	20	104
Al-25 wt% Si@Al_2_O_3_@Cu	36 μm		570			99.42, 570 °C	100	105
			518			*25*, *570 °C*		
						*41*, *518 °C*		

a *d*_core_: diameter of the core; *t*_shell_: thickness of the shell; *diameter of the shell; *T*_m_: melting temperature; *T*_c_: freezing temperature; *C*_p,l_: specific heat capacity; *λ*_l_: thermal conductivity (liquid); Δ*H*_m_: melting enthalpy. Italic value for Δ*H*_m_ is for Δ*H*_m_ after cycle test.

#### 3.2.2. Micro/nano encapsulation of high-melting-point metals and alloys

Owing to its superior stability and facile encapsulation, alumina is one of the most studied materials for the micro/nano encapsulation of high-melting-point metals and alloys (Table 4).

##### Metal-oxide nanoencapsulation

Navarrete *et al.* encapsulated commercial Al–Cu alloy nanoparticles in an Al_2_O_3_ layer that is formed naturally when the nanoparticles are exposed to oxygen.^96^ The encapsulated Al–Cu nanoparticles were added to the so-called solar salt (60 wt% NaNO_3_ and 40 wt% KNO_3_) to form nanofluids with improved TES performance. Thermal cycling analysis showed that the Al–Cu NEPCMs were chemically compatible with the solar salt and the oxide shell allowed for cycling up to 570 °C. Although the specific heat that contributes to SHS decreased with an increase in solids content, latent heat contributed by the enthalpy of fusion of Al–Cu NEPCMs increased the total TES to 17.8% within the same volume. Moreover, Al–Cu NEPCM incorporation increased the thermal conductivity of the nanofluids, which improved the heat-transfer performance of the solar salt-based HTF.

##### Metal-oxide microencapsulation

**Fig. 12:**
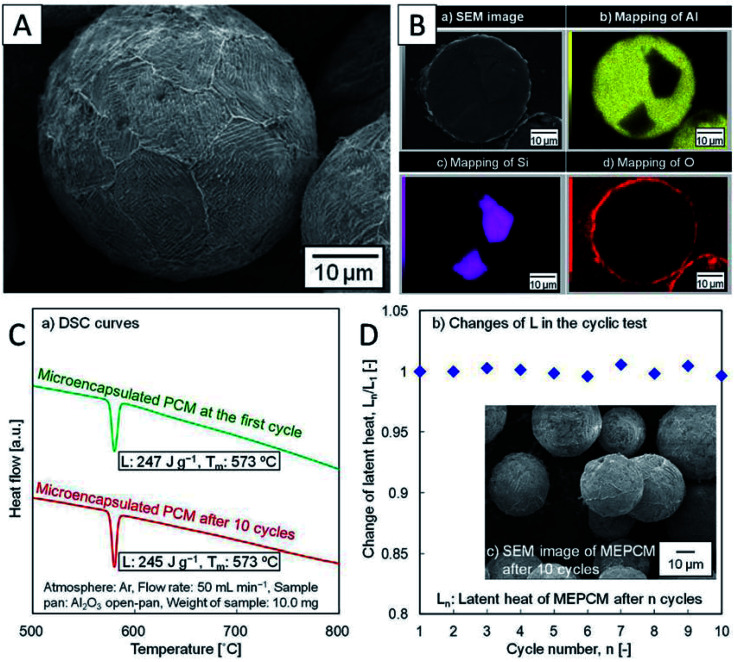
(A) SEM and (B) cross-section EDS elemental mapping images of Al–Si MEPCM. (C) DSC curves and (D) the changes in the latent heat of MEPCM during melt-freeze cyclic test in air. The inset is SEM of MEPCM after 10 melt-freeze cycles. Reproduced from ref. 58. Published by Springer Nature. Copyright 2015.

Nomura *et al.* first developed Al–Si alloy microsphere MEPCMs covered by α-Al_2_O_3_ shells (Fig. 12A and B) through a two-step procedure.^58^ In the first step, boehmite treatment was conducted to form an AlOOH shell on Al-25 wt% Si alloy microparticles. This was followed by heat-oxidation treatment of the resulting particles in an O_2_ atmosphere to transform the AlOOH shell into a more stable α-Al_2_O_3_ shell.^58^ The spherical Al-25 wt% Si microparticles (36.3 μm in diameter), which were used as raw materials, were produced by spinning-disk atomisation. This Al–Si-based MEPCM exhibited phase change at 573 °C with a latent heat of 247 J g^−1^ (Fig. 12C). In addition, melt-freeze testing for 10 cycles in air illustrated the protective effect of the encapsulating layer (Fig. 12D). In another study, Nomura *et al.* reported that in Al–Si alloy MEPCMs, voids inside the alloy core allowed for volume expansion of the PCMs during solid–liquid phase transition.^41^ In addition, the MEPCM exhibited excellent durability for up to 300 heating–cooling cycles in an O_2_ atmosphere and hence can be used in next-generation LHS-based high-temperature TES and transportation systems.

Furthermore, to understand the impact of Si content in the Si–Al alloy cores of the MEPCMs on their thermal storage properties, Nomura *et al.* examined MEPCMs with four different Al–Si compositions (Al-12 wt% Si, Al-17 wt% Si, Al-20 wt% Si, and Al-30 wt% Si).^97^ Their results revealed that the TES capacity of the MEPCMs increased and the heat-oxidation temperature decreased with a decrease in Si content near the eutectic composition. This is because when the alloy composition is different from the eutectic composition, the fraction of eutectic phase/primary Si solid in the MEPCM depends on Si content and a greater proportion of the primary Si phase reduces heat capacity. In addition, larger supercooling was observed in samples with higher Si content.

**Fig. 13:**
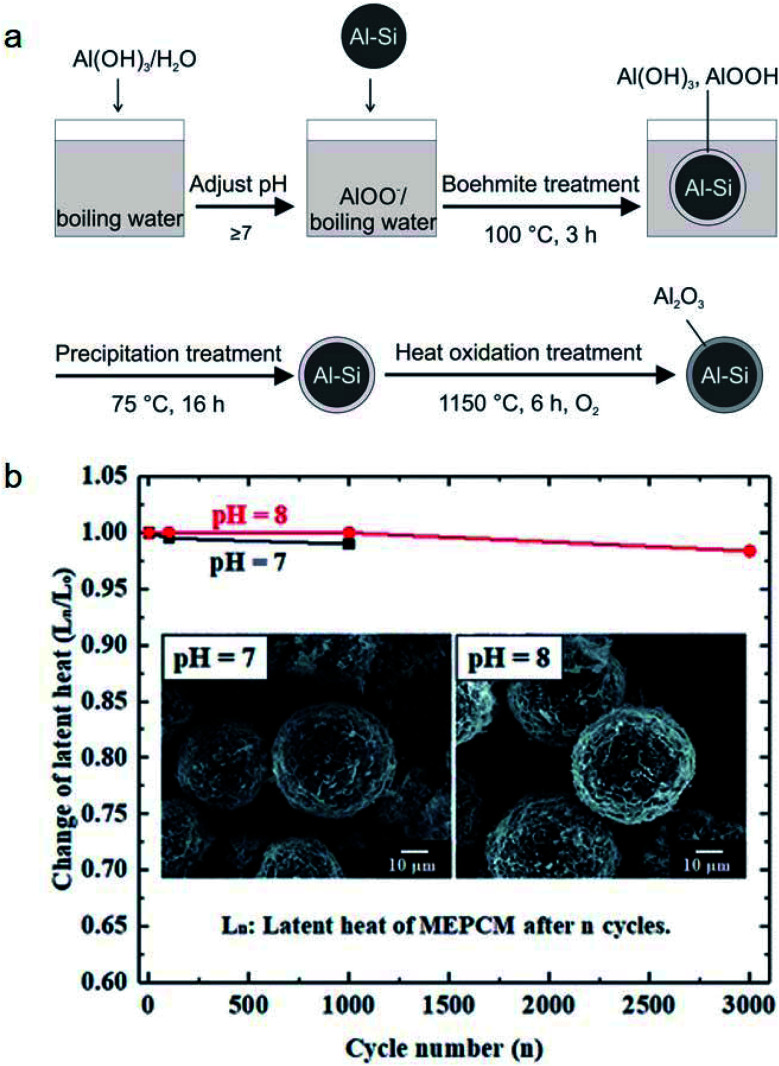
(a) Preparation of Al–Si/Al_2_O_3_ core/shell MEPCM particles. (b) Evolution of latent heat of MEPCMs for 3000 melting–solidification cycles. Inset shows the SEM images of the MEPCMs after 1000 melting–solidification cycles. Reproduced from ref. 98 with permission of The Royal Society of Chemistry, Copyright 2018.

It was found that leakage from MEPCMs occurred after 300 melt-freeze cycles owing to the non-compact, non-uniform, and cracked Al_2_O_3_ shells.^41,58,97^ To improve the shell stability of MEPCMs in high-temperature conditions, Nomura *et al.* developed Al–Si alloy MEPCM microspheres with high-temperature stability and good cycling durability using a 3-step method (Fig. 13a) consisting of (1) boehmite treatment of Al–Si microparticles at pH 8, (2) additional Al(OH)_3_ precipitation on the surface of Al–Si microparticles, and (3) heat oxidation in an O_2_ atmosphere. The newly formed boehmite and precipitation pre-treatment resulted in the formation of a thick and compact Al_2_O_3_ shell containing small α-Al_2_O_3_ and θ-Al_2_O_3_ grains. This resulted in good durability of Al–Si alloy MEPCMs over 3000 melt-freeze cycles in the air (Fig. 13b) by dispersing thermal stresses at high temperatures and restraining crack propagation in the Al_2_O_3_ shell.^98^

Apart from Al–Si-based NEPCMs, Nomura *et al.* also studied only Al (*T*_m_ = 660 °C) as the PCM to prepare core–shell MEPCMs composed of a stable α-Al_2_O_3_ shell and an Al core using the same two-step method as that used for Al–Si alloy MEPCMs. The heat-storage capacity of the Al MEPCMs decreased (273–301 J g^−1^) while the repetition durability improved with an increase in heat-oxidation temperature.^99^

**Fig. 14:**
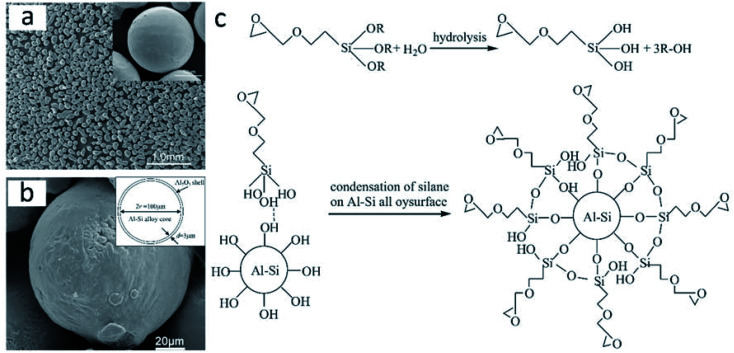
Inorganic microencapsulated core–shell structure of Al–Si alloy microparticles with silane coupling agent. SEM images of Al–Si alloy microparticles (a) after surface modification and (b) after surface modification and heating at 400 °C. The inset figure in (b) shows the sketch of Al–Si/Al_2_O_3_ core/shell particles. (c) Reaction mechanism of the coating process. Reproduced from ref. 100 with permission of Elsevier, Copyright 2014.

Using a sol–gel process based on the modification of a silane coupling agent (SCA), He *et al.* prepared Al–Si/Al_2_O_3_ core–shell microparticles with a dense α-Al_2_O_3_ shell (3–5 μm thickness; Fig. 14a).^100^ Commercial Al–Si eutectic alloy microparticles were washed with ethanol, followed by surface modification with SCA, and finally, treated with alumina sols. The obtained Al–Si/Al_2_O_3_ core–shell microparticles exhibited a latent heat of 307.21 kJ kg^−1^. SCA on the surface of Al–Si alloy microparticles played a key role in microencapsulation by promoting the condensation reaction between boehmite sols and silanol groups (Fig. 14b) to yield a dense Al_2_O_3_ shell. Fig. 14c shows the mechanism of the silane coupling agent treatment to Al–Si alloy. The researchers also studied the structural and phase-change characteristics of Al–Si/Al_2_O_3_ core–shell systems during melt-freeze cycling from room temperature to 1000 °C.^101^ The latent heat of the Al–Si/Al_2_O_3_ core–shell microparticles reduced to 271.90 kJ kg^−1^ after 20 melt-freeze cycles. The ruptured structure obtained at the end of the cycling period was attributed to the mismatch in the thermal stresses of the core and shell; it has been suggested that the presence of cracks at the core–shell interface can release thermal stresses during cycling and thus preserve the core–shell structure.

He *et al.* compared two methods for the synthesis of inorganic microencapsulated core–shell Al–Si alloy microparticles using the sol–gel process. These are (1) a pre-oxidation process and (2) modification of the original Al–Si alloy microparticles with an SCA.^102^ They found that both methods could be used for microencapsulation and resulted in a stable and dense α-Al_2_O_3_ shell. The pre-oxidation process usually generated a nonuniform shell, while modification with SCA resulted in more uniform microencapsulation. Moreover, according to zeta-potential measurements, both methods change the surface electric behaviour of Al–Si alloy microparticles; modification with SCA was more likely to result in the absorption of alumina sols on the surfaces, thus rendering thicker and more uniform Al_2_O_3_ shells.

**Fig. 15:**
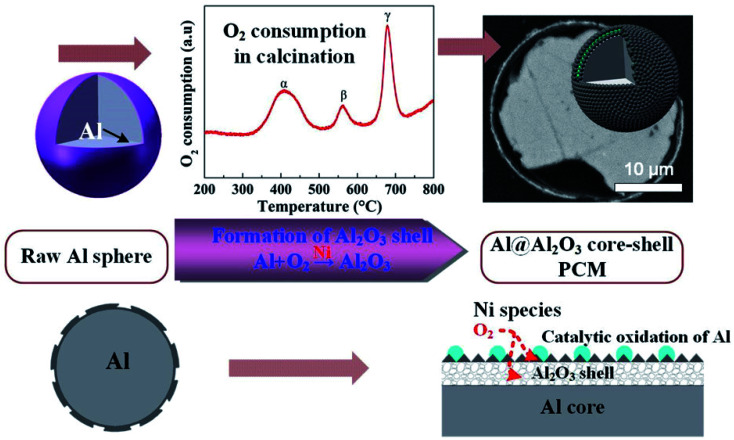
Synthesis of Al@Al_2_O_3_ microcapsule for high-temperature TES.^103^ Reproduced from ref. 103 with permission of The American Chemical Society, Copyright 2018.

Li *et al.* reported the microencapsulation of Al microspheres *via* an induced oxidation method.^103^ Nano-Ni species loaded on the surfaces of Al microspheres acted as catalysts to accelerate the oxidation of surface Al layer in air. Compared to unmodified Al, the oxidation activation energy (149–156 kJ mol^−1^) of nano-Ni-modified Al was much lower. By monitoring oxygen consumption and exothermic changes during the oxidation process, the researchers found that a 3-step oxidation of surface Al resulted in a layered Al_2_O_3_ shell (Fig. 15). Along with voids in the core, the layered Al_2_O_3_ shell helped in improving the elasticity of the core–shell structure, thus enhancing the thermal stability of the system during melt-freeze cycling.

Carbon may be used for surface modification to increase the thermal conductivity of metal-oxide encapsulated metal PCMs. Tian *et al.* used an *in situ* methane-decomposition method to deposit carbon on Al@Al_2_O_3_ microspheres.^104^ This method improved both the thermal conductivity and thermal stability of PCM particles.

##### Metal microencapsulation

**Fig. 16:**
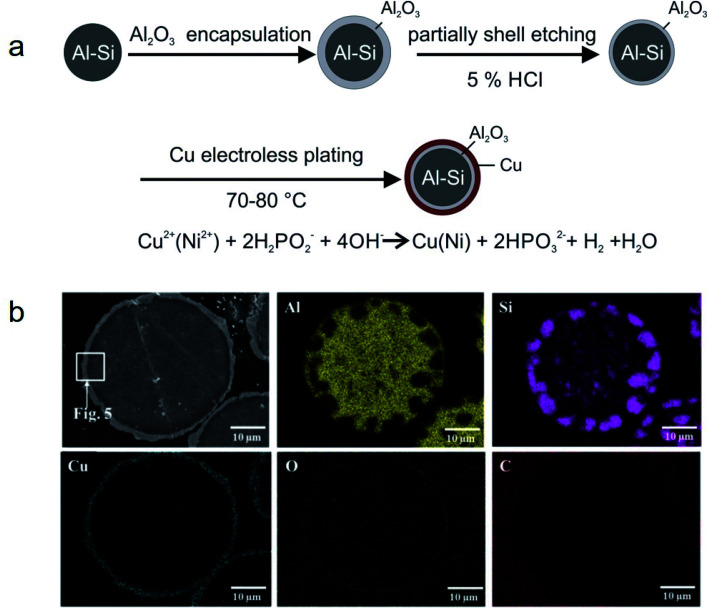
(a) Synthesis method to obtain Al-25 wt% Si@Al_2_O_3_@Cu MEPCMs for high temperature TES. (b) EDS elemental mapping on the cross-section of the resulting MEPCMs. Reproduced from ref. 105 with permission of Elsevier, Copyright 2019.

Owing to their good ductility, high thermal conductivity, and high melting temperature, some metals are good candidates as encapsulation materials for high-melting-point metal/alloy PCMs. Nomura and co-workers synthesised MEPCMs with spherical Al-25 wt% Si cores (average diameter of 36 μm) and Al_2_O_3_@Cu double-layered shells, as shown in Fig. 16a.^105^ The Al-25 wt% Si microparticles covered with Al_2_O_3_ shell were prepared by boehmite treatment followed by heating at 500 °C. Subsequently, HCl was used to etch the Al_2_O_3_-covered microparticles and electroless plating of a Cu layer was carried out to form multi-layered MEPCMs (Fig. 16b). The core–shell Al-25 wt% Si@Al_2_O_3_@Cu particles exhibited a low breakage ratio of ∼1.7% after 100 melt-freeze cycles.

Overall, Al_2_O_3_ and SiO_2_ are the most studied shell materials for encapsulation of the high-melting-point metal and alloy PCMs.^96–105^ Thermal annealing is often required to obtain dense and crystalline shells, which are desired for cycle stability. The core PCM particle for high-temperature PCMs is mostly in the micrometre scale.^96–105^ For high-melting-point metallic PCMs, a high value of LHS can be compromised for high cycle stability (oxidation of the core, shell thickness, shell materials).^98,105^ Further, there is still open room for nano encapsulation of high-melting-point metal and alloy PCMs.

### 3.3. Other applications of encapsulated metal and alloy PCMs

**Fig. 17:**
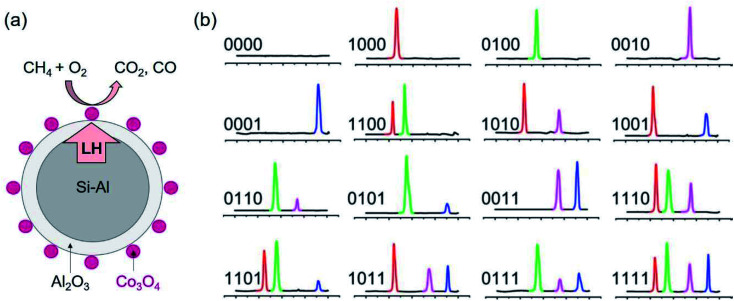
(a) Co_3_O_4_ deposited on the surface of Si–Al@Al_2_O_3_ PCM. The latent heat of PCM supplies to the catalytic reaction to burn methane on Co_3_O_4_ catalyst. (b) DSC curve of thermal barcodes.^111^ The scale (left to right) is from 100 to 300 °C. The presence of melting peaks of In, Pb–Sn, Sn and Bi at 156, 183, 232, 271 °C is used for barcoding. Reproduced from ref. 111 with permission of the American Institute of Physics, Copyright 2009.

Aside from applications in TES, the encapsulated metal and alloy PCMs have been utilised for catalysis, sensing and barcoding.^106–112^ Li *et al.* proposed an idea of using the latent heat of the core PCMs to manage the catalytic reaction of the catalysts on the surface of PCMs.^106^ The encapsulated metal PCM coupled catalyst (SiAl@Al_2_O_3_ PCM/Co_3_O_4_ catalyst) has been proved to allow for the conversion of methane after turning off the heat source for a long time (Fig. 17a).^107^ The methane conversion efficiency on PCM/catalyst was enhanced compared to Co_3_O_4_ itself. This is a promising direction for heat waste recovery and applications.

Su and co-workers reported low-melting-point metal and alloy PCMs (Sn, In, Pb, *etc.*) coated with SiO_2_ for biomarker detection.^108–110^ In this application, the PCMs were captured by biomarkers of different concentrations and types. The melting enthalpy of the PCMs-biomarker was found highly sensitive to the concentration of a biomarker in a large concentration range.^110^ The method allows for quantitative analysis of the biomarker concentration. Further, Su and co-workers could detect multiple DNA biomarkers with high sensitivity.^108–110^

Also utilising the latent heat of SiO_2_ encapsulated metal and alloy PCMs of different phase change temperatures, Su and co-workers demonstrated the capability of barcoding.^111^ At each melting temperature (peak of melting) the measured latent heat is written as 1 if the heat flux is detectable and otherwise as 0. In this way, they proved the concept of coding using thermal properties of a mixture of PCMs of different metals or alloys with distinct melting points. Mixtures of 4 types of nanoparticle PCMs, that is, SiO_2_ encapsulated In, Pb–Sn, Sn and Bi PCMs with the peak melting of 156, 183, 232, 271 °C, respectively, are used for barcoding. This allows for 16 combinations, thus, 16 barcodes (Fig. 17b). Other emerging applications of PCMs have also been reviewed.^112^

## 4. Conclusions and prospective outlook

High-density TES using PCMs is required in many industrial applications such as waste-heat recovery and in the harvesting of solar thermal energy. In addition, PCMs enable target-oriented discharging and control over the thermal sink temperature by enabling a constant phase-change temperature. Metals and alloys are considered as promising PCMs owing to their high thermal conductivity and volumetric heat-storage density. Moreover, micro- and nano-sized PCMs can be easily transformed into various forms, such as powders, pastes, slurries, and nanofluids. In this article, we reviewed recent research on micro- and nano-encapsulated metal PCMs for TES and highlighted the development of encapsulation approaches for metal and alloy PCMs with low and high melting points at micro- and nano-scales to achieve excellent thermal properties.

Most of the studies on the micro- and nano-encapsulation of metal and alloy PCMs are focused on preventing changes in PCM structure and protecting them from external environments. In addition, single-layer or single-shell encapsulation has been extensively studied. With the development of modern material chemistry, novel encapsulation structures, such as multi-shell structures and yolk–shell structures, can be finely designed for PCMs to achieve structural stability and high thermal storage performance. This aspect of micro- and nano-encapsulated metal PCMs is poorly explored, but offers great potential for new applications of these materials in thermal energy utilisation. One example is using structurally designed micro- or nano-encapsulated PCMs, which can not only deliver temperature maintenance but also provide venues for catalytic reactions.

## Author contributions

SZ composed the initial draft with consultation with MTN and TY. MTN and TY drew illustration and data presentation. MTN and TY revised the draft and completed the manuscript. All authors reviewed the manuscript. TY conducted overall management.

## Conflicts of interest

There is no conflict of interest to declare.

## Supplementary Material
